# Recent Developments of High-Resolution Chemical Imaging Systems Based on Light-Addressable Potentiometric Sensors (LAPSs)

**DOI:** 10.3390/s19194294

**Published:** 2019-10-03

**Authors:** Tao Liang, Yong Qiu, Ying Gan, Jiadi Sun, Shuqi Zhou, Hao Wan, Ping Wang

**Affiliations:** 1 Biosensor National Special Laboratory, Key Laboratory of Biomedical Engineering of Ministry of Education, Department of Biomedical Engineering, Zhejiang University, Hangzhou 310027, China; cooltao@zju.edu.cn (T.L.); zjubme_qy@zju.edu.cn (Y.Q.); ganying@zju.edu.cn (Y.G.); 3130102231@zju.edu.cn (J.S.); deardme@zju.edu.cn (S.Z.); 2 State Key Laboratory of Transducer Technology, Shanghai 200050, China

**Keywords:** LAPS, chemical imaging, spatial and temporal resolution, semiconductor, microfluidics

## Abstract

A light-addressable potentiometric sensor (LAPS) is a semiconductor electrochemical sensor based on the field-effect which detects the variation of the Nernst potential on the sensor surface, and the measurement area is defined by illumination. Thanks to its light-addressability feature, an LAPS-based chemical imaging sensor system can be developed, which can visualize the two-dimensional distribution of chemical species on the sensor surface. This sensor system has been used for the analysis of reactions and diffusions in various biochemical samples. In this review, the LAPS system set-up, including the sensor construction, sensing and substrate materials, modulated light and various measurement modes of the sensor systems are described. The recently developed technologies and the affecting factors, especially regarding the spatial resolution and temporal resolution are discussed and summarized, and the advantages and limitations of these technologies are illustrated. Finally, the further applications of LAPS-based chemical imaging sensors are discussed, where the combination with microfluidic devices is promising.

## 1. Introduction

**Figure 1:**
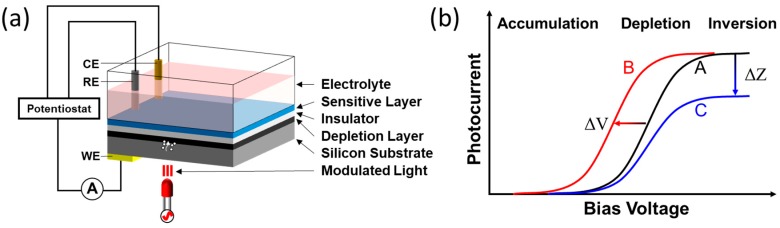
(**a**) Schematic of a typical LAPS set-up; (**b**) I-V curves representing the potential shift (LAPS) and impedance changes (SPIM).

The light-addressable potentiometric sensor (LAPS) [1,2,3,4,5,6] is a spatially resolved biochemical sensor based on the field-effect with an electrolyte-insulator-semiconductor (EIS) structure [7,8,9,10,11], in which the sensing surface of the insulating layer is in contact with the analyte solution. It shares the same structure as ion-selective field-effect transistors (ISFETs) and EIS capacitive sensors. The ISFET detects variation of the source–drain conductance, while the EIS capacitive sensor and LAPS detect the variation of the capacitance of the depletion layer, and the thickness of which responds to the potential of the sensing surface, thereby obtaining the analyte concentration. The characteristics of these field-effect devices have been compared and summarized systematically [12,13,14,15]. LAPS is developed from the combination of scanned light pulse technique (SLPT) method and EIS structures [6]. Figure 1a displays the schematic of a typical LAPS set-up. A standard set-up usually applies a three-electrode system, including a reference electrode (RE) such as Ag/AgCl, a counter electrode such as platinum wire, and a semiconductor substrate (silicon bulk) as a working electrode (WE). An ohmic contact is formed on the back of the semiconductor substrate to connect with the WE. The depletion layer forms at the semiconductor/insulator interface when a DC bias voltage is applied between the electrolyte and the semiconductor. After illuminating a frequency-modulated light at the top or bottom of the sensor, hole–electron pairs are generated. Then these photocarriers will be separated in the electric field and generate AC photocurrent, although some of them may recombine. This AC photocurrent has the same frequency as the modulated light and can be detected by the peripheral circuit.

LAPS sensors record local signals with spatial resolution because the photocurrent is generated only in the illuminated area, thereby achieving chemical imaging with a scanning light spot. This feature can not only measure local surface potential changes, but also the local impedance. The latter application is termed scanning photo-induced impedance microscopy (SPIM) [16,17,18]. The photocurrent-voltage (I–V) curves are shown in Figure 1b, LAPS detects the surface potential changes by recording the shift in the depletion region of the I-V curve along the voltage axis (shift of curve A to B), while SPIM measures the impedance changes by detecting the change of saturation current value in the inversion region (shift of curve A to C).

Since the first report in 1988 by Hafeman et al. [1], LAPS has been developed widely for sensing and imaging of (bio)chemical species [19,20,21,22,23,24] and living cells [25,26]. Such type of LAPS was initially termed pH-imaging sensor, as it first achieved the visualization of pH distribution [27,28,29,30,31,32,33]. After that, chemical imaging sensor was introduced to refer to the sensors that visualize the distribution of other chemical species, such as the enzyme [34], microcapsules [35] and ion diffusion [36,37].

The LAPS chemical imaging sensor has the following features and advantages. In contrast to the conventional electrode/sensor array, LAPS has no multi-pixel structure, and the pixel layout is defined by the scanning light, so the imaging region can be flexibly adjusted according to demand. Without the limitation of the pixel number, the image resolution is mainly determined by light spot size and scanning step distance. Besides, no additional wires are required, regardless of the imaging area and resolution which simplifies the structure of the circuit, and makes it durable for long-term use. Finally, LAPS can be integrated easily with microfluidic devices due to the flatness and uniformity of the sensing surface, which greatly increases the potential of LAPS for biomedical and micro liquid detection applications.

In recent years, various aspects of LAPS applications, including chemical species detection and surface modification have been comprehensively reviewed. Herein, we discuss and focus on the most advanced technologies of high-resolution imaging in the spatial and temporal aspects of LAPS-based chemical imaging sensor systems. The sensor system set-up, including the sensor chip construction, materials for sensing layer and substrate, modulated light property and various measurement modes are described and summarized. The prospects of future applications combined with microfluidic devices are discussed.

## 2. Measurement System Set-Up

The LAPS system for chemical imaging consists of a LAPS chip, a modulated light source, a mechanical device to move sensor plate or the position of the light spot to achieve the scanning. It also includes peripheral measurement circuit, electrochemical set-up like the three-electrode system, and PC software. The commonly used set-ups are discussed separately below.

### 2.1. Sensor Construction and Sensing Materials

A typical LAPS chip has a sensing layer/insulator/semiconductor substrate structure. The semiconductor is usually made of silicon bulk, covered with a thermally oxidized SiO_2_ layer. In order to reduce the recombination of photocarriers and improve spatial resolution, the silicon bulk can be replaced with thin silicon films (thinned silicon substrate [38,39,40], silicon on insulator (SOI) [18], amorphous silicon (a-Si) [41,42,43,44] and silicon on sapphire (SOS) [45,46]) or other semiconductor thin layers deposited on transparent substrates (GaAs [47], GaN [48], In-Ga-Zn oxide (IGZO) [49] and indium tin oxide (ITO) [50]). The sensing material such as Si_3_N_4_ [1,28,38,39,51], Al_2_O_3_ [52,53,54], Ta_2_O_5_ [51], HfO_2_ [55,56,57], NbO_x_ [40,58,59], SnO_x_ [60], self-assembled organic monolayer (SAM) [61,62] and nanofibers [63,64] can be used for pH sensing. Other chemical species can be also detected by modifying the sensing surface with appropriate materials. The deposition of chalcogenide glass films [65,66,67,68,69] can detect metal ions such as Pb^2+^, Fe^3+^, Cd^2+^ and Cr^6+^. The detection of inorganic salt ions (e.g., K^+^, Ca^2+^, Na^+^, Mg^2+^, Zn^2+^, Li^+^, NO_3_^−^, CO_3_^2−^, and SO_4_^2−^) can be achieved by depositing polymer membranes carrying ionophores such as PVC membranes [53,70,71,72,73,74,75,76], silicone rubber membranes [77,78,79,80,81] and photocurable membranes [82]. Immobilization of enzymes [83,84,85,86,87,88,89,90], antibodies [23,91,92] or DNA [93,94,95,96] on the sensing layer enables the detection of biomolecules such as glucose, urea, penicillin G, antigens, immunoglobulins G (IgG) and target DNA, etc. The sensing materials and target species have been systematically summarized [20,21,22].

### 2.2. Modulated Light

The modulated light illuminating the LAPS chip is usually sinusoidally modulated with a frequency in the range of 50 Hz to 100 kHz [97,98,99]. It is necessary to use the modulated light with a photon energy greater than the band gap. For silicon-based chips, visible or near-infrared light is commonly used. In terms of spatial resolution, according to the Rayleigh criterion, the resolution can be improved by reducing the light wavelength [45,61]. Regarding the illumination direction of the modulated light, there are two kinds of settings: Frontside and backside illuminations [97,100,101]. The backside illumination requires photocarriers to diffuse across the semiconductor substrate, which causes more recombination of the carriers and weakening the photocurrent signal. The long lateral diffusion distance will also reduce the spatial resolution. The frontside illumination directly generates photocarriers in depletion layer, and it has the advantages of larger signal amplitude and higher spatial resolution. However, the light needs to pass through the solution, specimen or cells, the scattering and absorption will disturb the light uniformity [102]. Therefore, the backside illumination is more practical and often applied.

### 2.3. Measurement Modes

LAPS is a kind of potentiometric sensor based on the field-effect, in which the sensing surface potential responds to the analyte concentration. The specific adsorption of the sensitive layer to the analyte changes the sensor surface potential. This sensing mechanism is essentially the same as ISFET and has already been explained systematically [9,103,104,105,106]. When the surface potential changes in response to the analyte concentration, the I-V curve also shifts accordingly. This change can be quantified by measuring the horizontal shift of the inflection point (that is calculating the zero-crossing point in the second derivative of the curve [3,28]). Then we can obtain the calibration curve between the inflection point voltage and the analyte concentration to achieve the measurement purpose. In most applications, we only need the changes of bias voltage at the inflection point. Acquiring the complete I–V curves at all pixels is too time consuming, especially in imaging applications. In order to meet different needs, the following measurement modes are usually used in different applications. Their pros and cons are summarized in Table 1.

#### 2.3.1. Constant-Voltage Mode

The method of obtaining the I-V curve of LAPS is to apply a scan bias voltage and then record the change in photocurrent with voltage. If there is no need to get a complete curve, we can just fix a constant bias and then measure the corresponding photocurrent. This constant-voltage mode (also called constant-bias mode) is fast and suited for multi-pixel imaging, thus it is usually used in the chemical imaging applications. As the surface potential of the sensor changes with the analyte concentration, the obtained photocurrent can be converted into the bias voltage by assuming a linear slope of the transition part of the I-V curve [27]. The voltage near the inflection point is usually chosen as the fixed bias, where the linearity and sensitivity is relatively good [107]. The constant-voltage mode has been used to obtain the pH image of *Saccharomyces cerevisiae* colonies [28] and to investigate the spatial resolution of LAPS [101]. However, the photocurrent variation of the constant-voltage mode is limited inside the transition part of the I–V curve. If the analyte concentration varies too large, the photocurrent will be saturated. Other than that, the assumed slope may cause some potential errors [107].

#### 2.3.2. Constant-Current Mode

Different from the constant-voltage mode, the constant-current mode chooses a constant current and then recording the change of the applied bias voltage, which requires a feedback loop adjusting the bias to maintain the photocurrent value in constant [4,107]. The results of this mode are more accurate because the recorded bias voltage change directly reflects the change of the analyte concentration, and does not need to be converted by the assumed slope, thereby avoiding some potential errors. In addition, there is no limit to the detection range, which allows the constant-current mode to measure larger analyte concentration variation. One constant current value can be set for all pixels of the ideal imaging sensor. However, general cases usually require two scans [21] since the chemical imaging sensor is not spatially uniform. The first scan records the initial photocurrent values of all pixels, and the second scan reproduces the photocurrent at each pixel. In this mode, an additional time is required until the sensor capacitance is charged when the bias voltage is updated. Therefore, the constant-current mode is more time consuming [38] and it is often used in analyte sensing applications rather than chemical imaging.

#### 2.3.3. Potential-Tracking Mode

In order to improve the accuracy of the results without sacrificing measurement time, Miyamoto et al. [27] proposed a new data acquisition method, namely the potential-tracking mode. In this mode, dozens of bias voltages are selected and the corresponding photocurrent values are recorded at each pixel. Then the entire I-V curve can be reconstructed by curve-fitting. Compared to measuring the complete I-V curve at each pixel, this mode can also obtain the shift of the entire I-V curve with shorter measurement time. Contrast with the constant-voltage mode, the proposed potential-tracking mode is able to measure a larger variation of analyte concentration, and the shift of I-V curve can more accurately reflect the variation of the analyte concentration. However, the potential-tracking mode also requires additional charging time to accommodate the new bias voltage, and requires an additional step of curve-fitting during the data processing.

#### 2.3.4. Phase Mode

For LAPS, the semiconductor substrate absorbs photon energy to generate hole-electron pairs, thereby the amplitude of sensor signal will be significantly affected by fluctuations in light intensity and the defects of semiconductor substrate [33,108]. In order to achieve accurate measurement, the phase-mode [109] was proposed to eliminate these effects. This mode detects the phase variation of the photocurrent, instead of the amplitude as in the common measurement mode. The AC photocurrent and the modulation signal are simultaneously recorded and then the phase difference between them are calculated. The phase-voltage curve shifts along the voltage axis in response to the analyte concentration variation, similar to the conventional I-V curve. The phase-mode is much less sensitive to the loss of photocarriers (caused by light intensity fluctuation and semiconductor defects), which contributes to the improvement of chemical image uniformity. During chemical imaging, the phase variations are recorded under a constant bias voltage. Errors may be also caused by the assumed slope during the phase-voltage conversion.

#### 2.3.5. Pulse-Driven Mode

The pulse-driven mode [110] utilizes a pulse-modulated light to generate the photocurrent rather than a conventional continuously modulated light. The photocurrent is mainly generated by two parts: photocarriers generated in the depletion layer and the diffused photocarriers. The utilization of a short light pulse with a high intensity can effectively eliminate the influence of the diffused photocarriers, and the shorter integration time of light pulse contributes to the improvements of spatial resolution (by a factor of 6 or more) and the contrast of line scan (by a factor of 3 or more), but it also results in a lower signal-to-noise ratio (SNR). In the pulse-driven mode, the photocurrent signal is acquired using a charge amplifier based on a high-speed operational amplifier rather than a trans-impedance amplifier to follow the fast change resulting from a light pulse. The measurement in pulse-driven mode takes more time than continuous modulation modes, because it needs extra time intervals to release the carrier distribution within the semiconductor between single pulses.

## 3. Spatial Resolution

Spatial resolution is one of the most important features of a chemical imaging LAPS; it has been defined as the smallest size of the pattern or structure that can be resolved by LAPS [111]. Photoresist patterns are deposited onto the insulator and then the resolution can be determined as the distance for achieving a photocurrent drop when scanning from an area without photoresist to another area with photoresist, by calculating the full width at half-maximum (FWHM) of the first derivative of photocurrent response across the edge of photoresist pattern [45,61]. A metal gate has also been used to determine the spatial resolution by measuring the photocurrent decay outside the gate area [17,41].

As a LAPS chip has no isolation between neighboring pixels, the spatial resolution is mainly determined by the lateral diffusion of minority photocarriers out of the illuminated area in the semiconductor layer [19,97,112]. Improving the spatial resolution can be carried out in two aspects: semiconductor substrate and modulated light property.

### 3.1. Semiconductor Substrate

Theoretical calculations showed that the resolution is determined by the lateral diffusion length of photocarriers in the semiconductor substrate [99,100,102]. In single crystalline silicon substrate, the diffusion length can be hundreds of micrometers. High doping concentration will increase the recombination of photocarriers and reduce the diffusion length, obtaining a higher spatial resolution [100,101,113]. However, more recombination of photocarriers also reduces the photocurrent signal, resulting in a loss of sensitivity.

Another approach to improving the spatial resolution is using the semiconductor material with a shorter diffusion length, such as GaAs [47], GaN [48] and amorphous silicon (a-Si) [41,42,43,44]. The shorter diffusion length also means more loss of photocarriers, resulting in a smaller photocurrent signal [97], which requires the semiconductor substrate must be thin. A diffusion length of 3.1  µm was measured using an 8 μm epilayer of GaAs [47], and a resolution of a thin film a-Si deposited on a transparent glass substrate is in the submicron range [41] with a diffusion length of 120  nm [114].

The simplest and most effective method to improve the spatial resolution is reducing the thickness of the semiconductor substrate [29,31,97,101,111,112]. With a thicker substrate, the photocarriers will travel a longer distance to arrive the depletion layer, causing a larger lateral diffusion length and a decreased resolution. Nakao et al. thinned the silicon substrate from 300 to 100 µm and further to 20  µm, achieved spatial resolutions of 500, 200 and 10 µm [31]. However, a silicon substrate thinner than 100 μm is fragile, and cannot provide a good mechanical support for practical applications. This problem can be solved by using the device with a thin semiconductor layer such as silicon on insulator (SOI) or silicon on sapphire (SOS). The use of SOI with a 7 µm thick device layer obtained an effective photocarriers diffusion length of about 13 μm and the diffusion length of SOS with 1 μm thick silicon layer was 570 nm [17]. Photocarriers are generated only in the light spot area, not the entire semiconductor layer, resulting in the elimination of the effects caused by stray light and reflections at edges. A resolution of 5 μm was obtained with another SOS device with 0.5 μm thick silicon layer [111].

**Figure 2:**
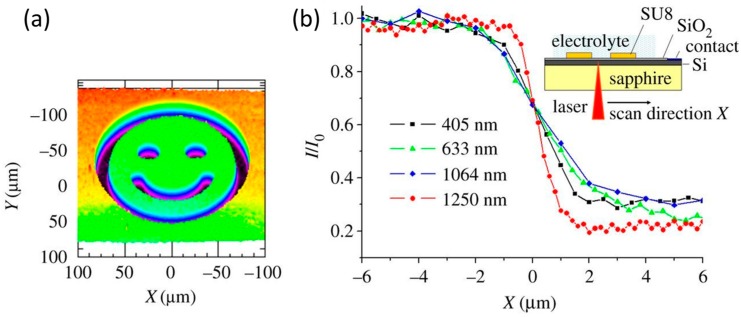
(**a**) Photocurrent image of the SU-8 pattern; (**b**) Normalized photocurrent curves scanning across the edge of the photoresist at different wavelengths. Reprinted (adapted) with permission from Chen et al. [45] Copyright (2019) Elsevier.

A transparent substrate such as sapphire can provide good mechanical support for a thin semiconductor layer, and the excellent light transmittance can achieve high spatial resolution in the case of backside illumination. Since it is difficult to grow a conventional thermal oxide layer on SOS [20], the insulating layer can be replaced with a good anodic oxide [45] or a self-assembled monolayer [61]. Currently, the best spatial resolution of LAPS/scanning photo-induced impedance microscopy (SPIM) with backside illumination is 0.8 µm, achieved using a SOS substrate with a 0.5 µm thick silicon layer and an anodic oxide [45]. An SU-8 pattern was fabricated on the insulator and the photocurrent image is shown in Figure 2a. Modulated lights with different wavelengths of 405, 633, 1064 and 1250 nm were used and obtained the spatial resolutions of 1.5, 2.2, 3.0 and 0.8 μm respectively. The normalized photocurrents curves scanning across the edge of the photoresist at these wavelengths are displayed in Figure 2b.

**Figure 3:**
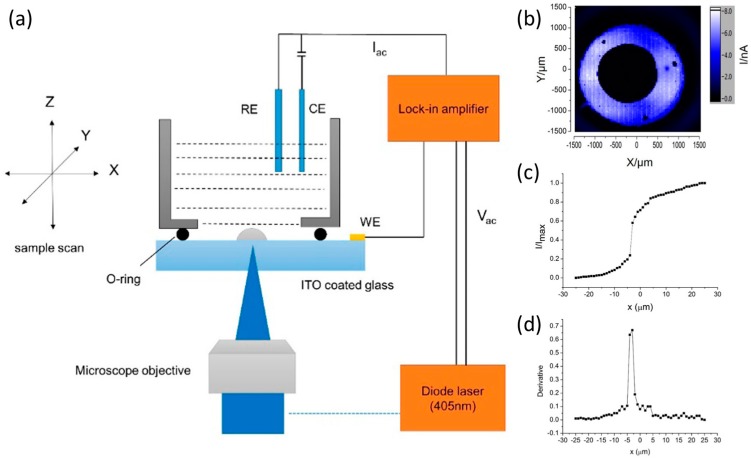
(**a**) Schematic of the LAPS set-up with ITO coated glass; (**b**) The LAPS image of the PMMA dot; (**c**) Normalized photocurrent-position curve scanning across the edge of PMMA dot; (**d**) the first derivative of (B) with the FWHM of 2.3 μm. Reprinted (adapted) with permission from Zhang et al. [50] Copyright (2019) American Chemical Society.

Although SOS has many advantages, the high cost limited its widespread application. In recent years, indium tin oxide (ITO) coated glass has been chosen as a new substrate for LAPS and SPIM as a bioelectronic taste sensor for bitter [115] or a high resolution imaging sensor [50] due to the features of low cost, robustness, stability and easy to be modified. The LAPS with ITO coated glass as the substrate has electrolyte–semiconductor (ES) structure without an insulator. The schematic of the LAPS set-up is shown in Figure 3a. The wavelength of the modulated light illuminated from back side is 405 nm. The verification of UV-vis spectrum and impedance spectrum proved that the excitation light wavelength is in the UV part and the blocking nature of the ITO-solution interface in the dark. Different from the conventional structure, the LAPS with ITO coated glass has no insulating layer, and the measured photocurrent signal is generated from the redox currents, which is affected by the pH dependent kinetics in the anodic oxidation process. The average pH sensitivity of this ITO coated glass LAPS is about 70 mV/pH, higher than the Nernstian theoretical value of 59 mV/pH. Although high sensitivity is good for sensors, this super Nernstian sensitivity is usually caused by multiple potential changes, and the selectivity of the ITO coated glass sensor has not been verified. Poly (methyl methacrylate) (PMMA) dots on the ITO surface wasre used to characterize the lateral resolution. Figure 3b shows the photocurrent image of the dot pattern. Due to the high impedance of PMMA, the photocurrent in polymer dot area decreased to nearly zero. To determine the lateral resolution, a line scanning across the edge of the dot was carried out at a step of 1 μm. The normalized photocurrent-position curve is shown in Figure 3c. The full width at half-maximum (FWHM) of the first derivative of photocurrent response across the edge is usually used to indicate the lateral resolution. As shown in Figure 3d, the FWHM (lateral resolution) is about 2.3 μm, close to the 1.5 μm resolution of the SOS at the wavelength of 405 nm. This indicates that the ITO coated glass can be a low-cost alternative material for SOS for high resolution imaging.

**Figure 4:**
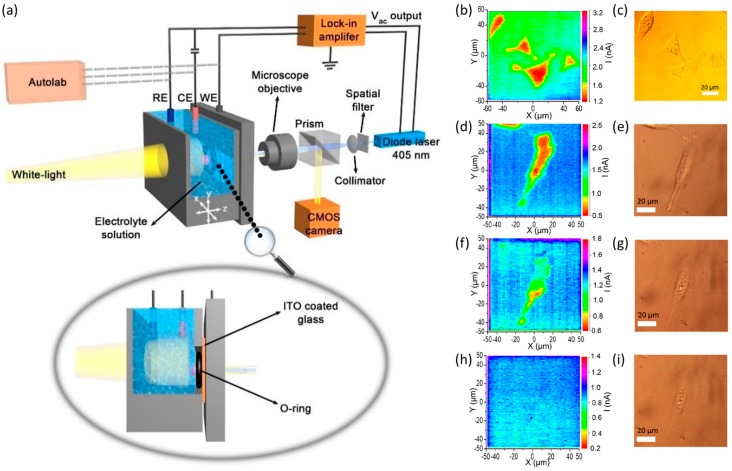
(**a**) Schematic of the PEIS set-up with ITO coated glass; (**b**) photocurrent image by PEIS and (**c**) optical image by camera of MG63 human osteosarcoma cells; (**d**) PEIS image and (**e**) optical image of hMSC before TX-100 addition; (**f**) PEIS image and (**g**) optical image after 30 min incubation with 0.04% TX-100 in DPBS; (**h**) PEIS image and (**i**) optical image after 200 min incubation with 0.04% TX-100 in DPBS. Reprinted from Wu et al. [116].

Recently, the LAPS with ITO coated glass substrate has been developed into a novel photoelectrochemical imaging system (PEIS) for single live cells imaging [116]. The mapping of surface charge of the cell bottom can be obtained with the set-up shown in Figure 4a, the photocurrents in this structure features the redox currents. The photocurrent image of MG63 human osteosarcoma cells by PEIS is shown in Figure 4b, while the optical image by CMOS camera is shown in Figure 4c, indicates a great correlation with the former. The photocurrent in the cell area was smaller, this may be caused by the negative charge of the cell surface. In addition, the process of cell lysis can be also monitored with PEIS. Human mesenchymal stem cells (hMSC) and 0.04% *v/v* TX-100 (0.68 mM) in Dulbecco’s Phosphate Buffered Saline (DPBS) was used for cell membrane permeabilization and cell lysis. Initially, the PEIS image (Figure 4d) and optical image (Figure 4e) without TX-100 shows a clear photocurrent distribution and the intact profile of cell membrane. 30 min after the TX-100 addition (Figure 4f,g), the cell has lost its intact form, the increased photocurrent in the cell area indicating the progressive solubilization of cell membrane. And after 200 min, the cell profile disappeared in the PEIS image (Figure 4h), indicating that the cell membrane has completely broken down. This is consistent with the optical image (Figure 4i) where only the residues of the cell remained. LAPS has already achieved the visualization of the defects recovery process in a cultured cell layer [25], now with the help of the abovementioned PEIS, the visualization extent of the metabolism and lysis can be accurate to the single cell level, which expands the application prospects of LAPS imaging.

### 3.2. Property of Modulated Light

In addition to the choice of semiconductor substrate, the modulated light property also affects the spatial resolution. Reducing the light spot size is a direct way to increase the spatial resolution. Nakao et al. [28] focused the modulated light spot to 1 μm size for the first time and obtained the pH distribution image of the *Saccharomyces cerevisiae* colonies. In previous reports, the effect of the light wavelength on the resolution has been investigated [31,45,61,100,113].

The use of infrared light can improve the spatial resolution. For the bulk silicon substrates with backside illumination, the light with long wavelength can reach deeper into the silicon and photocarriers will be generated nearer to the depletion layer. 10 μm test patterns can be resolved with an infrared light (the wavelength is 830 nm) illuminating a 20 μm silicon substrate [31], in which the penetration depth is 13 μm.

**Figure 5:**
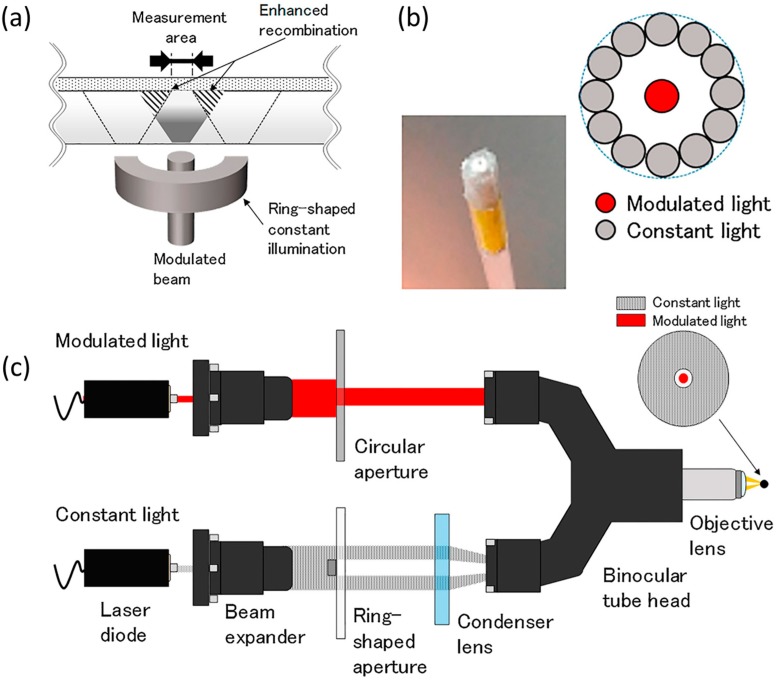
(**a**) Schematic of the LAPS with hybrid illumination; (**b**) Assembly of the ring-shaped bundled fiber-optics; (**c**) Schematic of the hybrid illumination set-up based on binocular tube optics. Reprinted (adapted) with permission from Miyamoto et al. [120] Copyright (2019) Elsevier.

To improve spatial resolution, an auxiliary illumination of constant light can be added around the modulated light [117,118,119,120]. As shown in Figure 5a, a ring-shaped constant illumination increases the photocarriers concentration, and the enhanced recombination will block the lateral diffusion of photocarriers induced by the modulated light. The effect was predicted by simulation [117], and the experiment [119] verified a resolution improvement from 92  µm without to 68  µm with the ring-shaped constant illumination by bundled fiber-optics (Figure 5b). Simulations [118] suggest that the resolution would be limited due to the enhanced recombination, causing a decrease of the photocurrent amplitude. And the spread of light after exiting the end of fiber-optics will blur the shapes of both the modulated light spot and the ring-shaped constant illumination. In order to further improve the resolution, the hybrid illumination system based on a binocular tube can be used instead of the bundled fiber-optics [120]. The binocular tube optics set-up is shown in Figure 5c and it can resolve a 100 µm pattern while the bundled fiber-optics cannot, indicating that the spatial resolution has been further improved.

The use of pulse-driven light [110] can also improve the spatial resolution, which has been discussed in Section 2.3.5 of this article. For the thin silicon substrates such as SOS [45,61], the penetration depth of the light is less important. The effect of wavelength on spatial resolution is mainly determined by the Rayleigh criterion: r=0.61λ/NA, where r is the resolution, λ is the wavelength and NA is the numerical aperture of the microscope objective. In the wavelength range of 405 nm to 1064 nm, the spatial resolution increases as the wavelength decreases. The best resolution is 1.5 µm at the wavelength of 405 nm. Further improvement of the resolution to 0.8 µm can be achieved based on the two-photon effect with a 1250 nm light. In this case, the photon energy of the modulated light was smaller than the energy bandgap of silicon, the photocarriers were generated under the two-photon effect only near the focused spot close to the depletion layer [17]. All the technical information about spatial resolution mentioned above is summarized in Table 2.

## 4. Temporal Resolution

A typical way of LAPS imaging is to use a single beam scan to readout the photocurrent values of each pixel one by one. The image acquisition time is given by multiplying the measurement time of each pixel by the number of pixels. In a typical set-up, the measurement time per pixel is millisecond level, and the acquisition time for one image is on the order of minutes at a resolution of 128 × 128 pixels, for example. In addition, the scanning speed of the mechanical stage also greatly limits the temporal resolution. The minute-level time consuming is acceptable for monitoring some slow processes, such as metal corrosion [121] and cell growth [25,30], but difficult for the fast processes such as chemical reactions and ion diffusion. Therefore, it is necessary to reduce the measurement time.

### 4.1. Single Modulated Light Without Mechanical Movement

**Figure 6:**
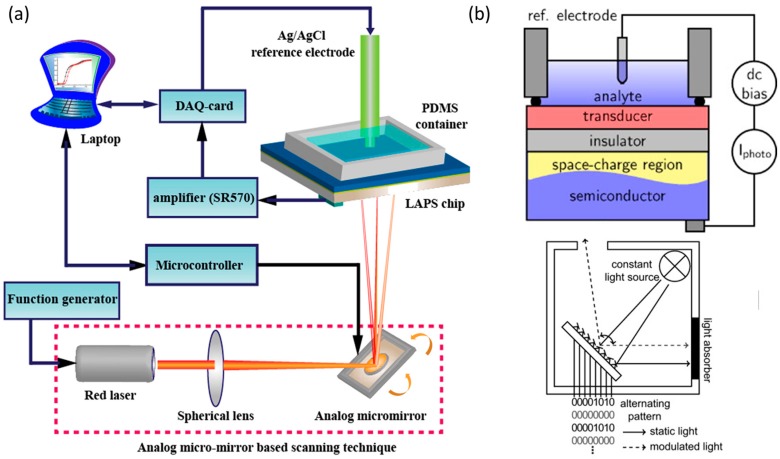
(**a**) Schematic of the LAPS imaging system based on analog micromirror. Reprinted from; (**b**) Schematic of the LAPS imaging system based on the DLP, the light is modulated by the fast switching mirrors and the position and size of the light spot can be adjusted as required. Reprinted (adapted) with permission from Das et al. [124] and Wagner et al. [125] Copyright (2019) Elsevier.

An analog micromirror based on micro-electro-mechanical systems (MEMS) has been used to replace the traditional mechanical stage [122,123,124]. The schematic of this set-up is shown in Figure 6a, the light spot can be moved and located precisely on the back of the sensor plate according to user requirements by PC software control. This set-up was able to acquire a chemical image with the resolution of 500 × 400 pixels within 40 s, and can also record the spatiotemporal change in pH distribution in KCl solution and buffer solution with a rate of 16 frames per second (fps) and a resolution of 10 × 8 pixels per frame. However, in this way, the angle of light illumination at each pixel is different, resulting in different spot sizes on the back of the sensor plate, which affects the uniformity of the image.

In order to completely avoid the effects of mechanical motion (including the rotation of the micromirror), digital micromirror device (DMD) based on the digital light processing (DLP) technology was implemented as the scanning light source for LAPS [125,126]. The system set-up is shown in Figure 6b. The modulated light is generated by the fast switching DMD, the size and shape of light spot can be easily defined according requirements without any modification of hardware. This set-up has 480 × 320 micromirrors, resulting in a maximum resolution of 153,600. The authors investigated a method that uses a big size spot to perform a low-resolution scan of the entire sensor surface (just a few seconds) firstly. After determining the region of interest (ROI), a small-sized spot is used to scan the local region with a high-resolution. This method is expected to combine the advantages of fast imaging and high-resolution scanning.

In addition, some commercial products suggest illuminating different spots on the LAPS sensor plate, such as a projector based on DLP [42] or liquid crystal [127], an OLED display [125,128]. All the devices are capable of addressing a large number of pixels without mechanical movements, and can customize the measurement areas freely. However, the OLED displays and projectors are designed for video applications, which require low refresh rates (typically 25 fps), so the modulation frequencies in these set-ups are lower than 1 kHz, resulting in a relatively long sampling time, limiting the future applications of fast imaging. Werner et al. developed a new driving method for OLED-LAPS [129], enabled to define the modulation frequencies between 1 kHz and 16 kHz. The increased frequency modulation light speeds up the measurement and reduces the acquisition time of image by a factor of 40 compared with ordinary OLED display [128].

### 4.2. Multi-Frequency Modulation Light Source Array

Another method for improving the temporal resolution is the utilization of light source array, illuminating multi pixels simultaneously with different modulated frequencies. The fast Fourier transform (FFT) algorithm is used to separate and reconstruct the signal for each frequency from the mixed signal. In order to successfully separate each signal in the frequency domain, it is worth noting that the step of modulation frequency must be larger than or equal to the inverse of the sampling time [21,130,131], and the highest frequency is required to be less than twice the lowest frequency to avoid the harmonic interference [19,131]. Zhang et al. proposed this method for the first time in 2001 [132], the LAPS sensor plate was illuminated simultaneously by 3 light spots at different pixels with the frequencies of 3.6, 3.8 and 4.0 kHz. Later, this method similar to the frequency-division multiplexing (FDM) has been applied for rapid imaging system and multi-sensor with multiple light sources. A light source array consists of 4 × 4 LED for multi-sensor was employed [68], and demonstrated the possibility of recording cell metabolism and ion detection simultaneously at multiple spots on one chip. Hu et al. [69] reported three parallel measurements using line-array focused laser for heavy metal detection. Miyamoto et al. [130] proposed a combination of light source array and the traditional mechanical scanning. A linear array of 16 LEDs with an interval of 3.6 mm was used at different frequencies, the scan speed of this linear LED array was 12.5 mm/s and obtain a image at a resolution of 16 pixels × 128 lines within 6.4 s. In this method, increasing the density of the LED array allows for more measurement spots, and higher resolution can be obtained at the same sampling time. Wagner et al. [133] applied a high-density multi-point LAPS based on a miniaturized vertical-cavity surface-emitting laser (VCSEL) array. It consisted of 12 VCSEL diodes with a pitch of 250 μm on a 3 mm single substrate, and the modulation frequencies of each diode were generated by the FPGA in the range of 3 kHz to 4.1 kHz, with a step of 100 Hz. This set-up could operate measurement spots simultaneously and reduce the overall measurement time.

**Figure 7:**
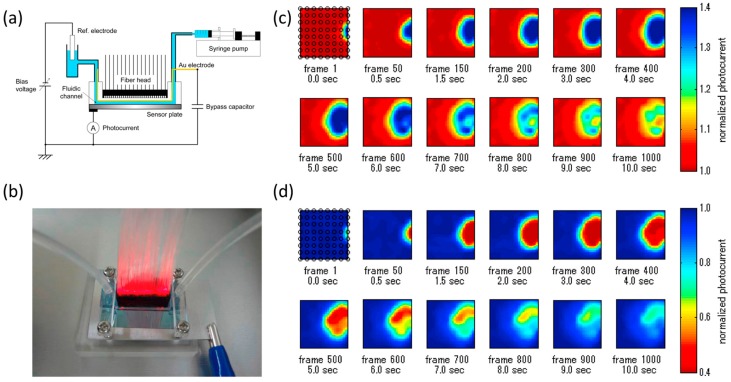
(**a**) Schematic of the LAPS with microfluidic channel set-up; (**b**) The 64 light beams guided by the optical fibers above the LAPS surface; (**c**) Injection of 10 mM NaOH and (**d**) 10 mM HCl into PBS solution. Reprinted (adapted) with permission from Miyamoto et al. [131] Copyright (2019) Elsevier.

For LAPS imaging applications, the high modulation frequency requires a lower sampling time, which contributes to the improvement of image speed. In the backside illumination LAPS, the highest modulation frequency is limited by the low-pass filtering characteristics of photocarriers diffusion across the semiconductor substrate [97]. The available modulation frequency can be therefore extended by using frontside illumination or a thinner semiconductor substrate, which can achieve a higher imaging rate. A front-side-illuminated LAPS with two-dimensional LED array was proposed [134]. With the sampling frequency of 400 kHz, the visualization of pH distribution could be recorded at the rate of 70 fps. After that, a high-speed chemical imaging system [131] based on 64 illumination spots guided by optical fibers was developed for dynamic pH measurement inside a microfluidic channel on the LAPS chip surface. Figure 7a shows the schematic of the LAPS with microfluidic channel, the light source array is above the LAPS and performs a liner scan. The photo of 64 light beams guided with optical fibers are shown in Figure 7b, the modulation frequencies are in the range of 6.4 kHz to 12.7 kHz with the step of 100 Hz. In the application of pH distribution visualization, the optical fibers were bundled in a layout of 8 × 8 with an interval of 1.5 mm, and an imaging area of 12 × 12 mm^2^ was acquired. The sampling frequency, the sampling number, and the sampling time were 400 kHz, 4000, and 10 ms respectively, which achieved a high-speed imaging at 100 fps. 10 mM NaOH and 10 mM HCl was respectively injected into the phosphate buffered saline (PBS), the frames of the spatiotemporal change in pH distribution are shown in Figure 7c,d, the changes in photocurrent correspond to the changes in pH. A temporary change in pH and subsequent recovery can be clearly observed. All the technical information about temporal resolution mentioned above is summarized in Table 3.

## 5. Integration with Microfluidic Devices

For some biomedical applications with small sample volumes, integrating the sensors with microfluidic devices is a promising strategy. The LAPS-based imaging sensor is a well-suited sensor for the combination with microfluidic devices [37,131,135] as the following advantages: Firstly, the LAPS chip surface is flat and uniform, which facilitates its fit with microfluidic channels [136] and microchambers [137] of any shape. The commonly used materials for the sensor chip (silicon oxynitride) and microfluidic channel devices (polydimethylsiloxane (PDMS)) can be easily bonded together by plasma treatment. Secondly, due to the addressability of LAPS, any position within the microfluidic channel is measurable [138], which contributes to the assays based on chemical imaging. Thirdly, thanks to the good breathability, biocompatibility and light transmission of PDMS materials, LAPS integrated with microfluidics can be used for biomedical measurements [139] and optical images can also be obtained under a microscope.

**Figure 8:**
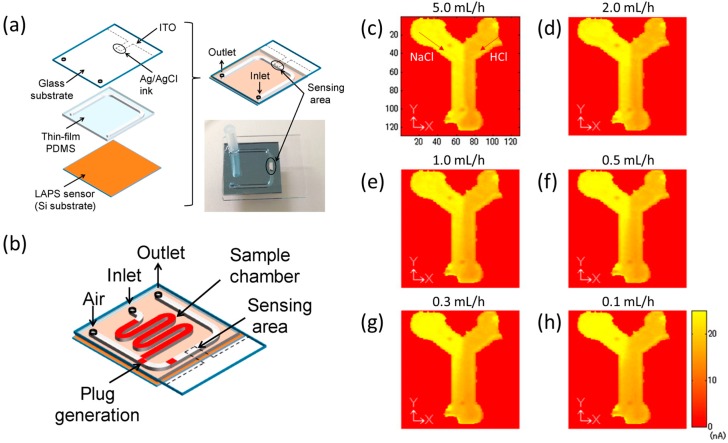
(**a**) Schematic of the microfluidic devices combined with LAPS; (**b**) Microfluidic channel design for plugs generation on LAPS chip. Reprinted from; Chemical images of the laminar flows in the Y-shaped channel under the injection rates from (**c**) 5.0 mL/h to **(h)** 0.1 mL/h. Reprinted (adapted) with permission from Miyamoto et al. [138] and Miyamoto et al. [37] Copyright (2019) Elsevier.

LAPS-based chemical imaging sensor can simultaneously analyze the chemical reaction and diffusion of the analyte in a microfluidic channel. Miyamoto et al. [138] proposed a plug-based microfluidic system combined with LAPS. The structure of the integrated system is shown in Figure 8a. The analyte solution was injected into the channel as a plug form with the volume of microliters. Because of the light-addressability, the plug could be monitored at any position and pneumatically controlled. Figure 8b shows the microfluidic channel design to generate plugs. The analyte solution in the sample chamber was divided into a series of plugs by the injected air and then passed through the sensing area.

The LAPS-based chemical imaging sensor has also been applied to investigate the concentration profile of ion diffusion in a microfluidic channel [37]. A Y-shaped microfluidic channel was fabricated on the LAPS chip surface, with the thickness and the width of 160 μm and 2 mm. 0.1 M NaCl and 0.1 M HCl were injected respectively into the left and the right branches of the Y-shaped channel, and the chemical image of laminar flows were obtained under the injection rates from 5 mL/h to 0.1 mL/h, as shown in Figure 8c–h. A boundary of the two streams can be clearly seen in Figure 8c because the contacting time is too short and there is no significant change of the pH distribution. As the flow rate decreasing, more ions diffusion across the interface of the two laminar flows can be observed, from which the diffusion coefficient can be calculated. Since the diffusion coefficient depends on the molecular weight of chemical species, this assay can be used as a mass spectrometric measurement of an unknown molecule.

## 6. Conclusions

Since LAPS was first proposed in 1988, it has been widely developed for biomedical and chemical studies. LAPS are suitable for detecting potential changes induced by changes in ion concentrations, and can also be applied for the measurement of local impedance (SPIM). The flat and uniform surface of LAPS chip facilitates the deposition of sensitive materials and the immobilization of specific identification elements, detections for metal and inorganic salt ions, biomolecules such as enzyme, antibody, glucose and DNA, etc. have been achieved so far.

The performance of LAPS chemical imaging has also enhanced over the past two decades. For high spatial resolution imaging, the LAPS substrate materials including thinned silicon, amorphous silicon (a-Si), and ultra-thin semiconductor layers on transparent substrates have been investigated. The effect of the modulated light property on spatial resolution has also been studied. The best spatial resolution reported to date is 0.8 µm obtained with a SOS device based on the two-photon effect. Based on the photoelectrochemical imaging system (PEIS) with ITO-LAPS, the visualization extent of the metabolism and lysis can be accurate to the single cell level, which expands the application prospects of LAPS imaging.

The use of a FDM-based multi-light source array is an effective method for high-speed imaging. Parallel measurement of multiple pixels allows signals of tens of thousands of pixels to be read out in one second. This high temporal resolution is able to measure the processes of fast chemical reactions such as ion diffusion. A variety of measurement modes were designed to meet different needs, and image calibration algorithms were developed to correct the non-uniformities of sensor chip.

For further application of LAPS, in addition to improving the sensor performance, the miniaturization multi-functionalization of the detection system is also important. It is a promising prospect that setting multiple measurement sites on the sensor surface and integrating with the microfluidic device to establish a “lab-on-a-chip”. On one hand, the volume of sample in microliter grade can reduce costs; on the other hand, it enables rapid and multi-parameter detection under controllable dynamic conditions with the emergence of novel designed microfluidic devices [140]. The integrated sensor system will be able to be applied in biomedical applications for a variety of purposes.

## Figures and Tables

**Table 1:** Pros and cons of LAPS measurement modes.

Measurement Modes	Pros	Cons	Reference
Constant-Voltage Mode	Rapid Measurement; Suitable for Multi-Pixel Imaging	Small Detection Range; Potential Conversion Rrrors	[28,101]
Constant-Current Mode	Unlimited Detection Range; Accurate Measurement	Necessary Feedback Loop; Long Measurement Time	[4,107]
Potential-Tracking Mode	Entire I-V Curves; Unlimited Detection Range; Relatively Accurate	Additional Curve-Fitting; Necessary Charging Time	[27]
Phase Mode	Good Robustness; Good Imaging Uniformity	Necessary Simultaneous Record of Photocurrent and Modulation Signal; Potential Conversion Errors	[109]
Pulse-Driven Mode	High Spatial Resolution; High Contrast of Line Scan	Low SNR; Long Measurement Time	[110]

**Table 2:** Technical information about improving the spatial resolution of LAPS.

Methods		Notes	Sensor Construction	Modulated Light Parameters ^1^	Spatial Resolution	Reference
Semiconductor Substrate Properties	High Doping Concentration	Simulation Results	50 nm SiO_2_/50 nm Si_3_N_4_/200 μm Si	Backside Illumination; λ = 800 nm, f = 5 kHz, P = 6 W/cm^2^, S = 20 μm	<30 μm	[100]
	Short Diffusion Length Materials	Semiconductor Material: GaAs	8 μm Pt/100 nm Anodic Oxide/8 μm Epilayer of GaAs	Frontside Illumination; λ = 780 nm, f = 10 kHz, P = 0.18 mW, S = 2.6 μm	3.1 μm	[47]
		Semiconductor Material: Amorphous Si	20~150 nm Metal Gate/50 nm Si_3_N_4_/30 nm SiO_2_/0.3~1.5 μm a-Si/Glass/500 nm Al/700 ZnO	Frontside Illumination; λ = 430 nm, P = 1 mW, S = 1.03 μm	<1 μm	[41]
	Thinned Semiconductor Substrate	Infrared Light	100 nm Au/Photoresist Pattern/100 nm Si_3_N_4_/50 nm SiO_2_/20 μm Si	Backside Illumination; λ = 830 nm, f = 1~10 kHz, S = ~1 μm	<10 μm	[31]
	Transparent Substrate with Thin Semiconductor Layer	Silicon on Sapphire	Photoresist Pattern/6.7 nm Anodic Oxide/0.5 μm Si/500 μm Sapphire/20 nm Cr/80 nm Au	Backside Illumination; λ = 405 nm, f = 1 kHz, P = 1 mW (Single Photon Effect)	1.5 μm	[45,61]
		ITO Coated Glass; No Insulator	PMMA dot/~140 nm ITO/500 μm Glass	Backside Illumination; λ = 405 nm, f = 10 Hz, S = ~1 μm	2.3 μm	[50,116]
Modulated Light Properties	Small Light Spot Size	Spot Size at Micron Level	100 nm Si_3_N_4_/50 nm SiO_2_/300 μm Si/AuSb	Backside Illumination; λ = 633 nm, f = 1~10 kHz, P = 10 mW, S = ~1 μm	<500 μm	[28]
	Infrared Light	Thin Silicon Substrate	100 nm Au/Photoresist Pattern/100 nm Si_3_N_4_/50 nm SiO_2_/20 μm Si	Backside Illumination; λ = 830 nm, f = 1~10 kHz, S = ~1 μm	< 10 μm	[31]
	Auxiliary Illumination	Ring-Shaped Constant Light	50 nm Si_3_N_4_/50 nm SiO_2_/200 μm Si/Au	Backside Illumination; λ = 832 nm, Modulated, P = 0.002 mW; λ = 832 nm, Constant, P = 0.1 mW	<68 μm	[119,120]
	Pulse-Driven Modulated Light	Charge Amplifier	40 nm Si_3_N_4_/40 nm SiO_2_/200 μm Si/Au	Backside Illumination; λ = 905 nm, t = 2 μs, P = 85 mW, S = ~1.1 μm	110 μm	[110]
	Two-Photon Effect	Silicon on Sapphire	Photoresist Pattern/6.7 nm Anodic Oxide/0.5 μm Si/500 μm Sapphire/20 nm Cr/80 nm Au	Backside Illumination; λ = 405 nm, f = 1 kHz, P = 1 mW (Two-Photon Effect)	0.8 μm	[45,61]

^1^ λ: Wavelength; f: Frequency; P: Power; t: Integration time; S: Light spot size.

**Table 3:** Technical information about improving the temporal resolution of LAPS.

Methods		Notes	Sensor Construction	Modulated Light Parameters ^1^	Temporal Resolution ^2^	Reference
Single Modulated Light Without Mechanical Movement	Analog Gimbal-Less Two-Axis Micromirror	Light Spot Movement by Angular Rotation	10 nm Si_3_N_4_/3 nm SiO_2_/500 μm Si	Backside Illumination; λ = 658 nm, f = 5~20 kHz, S = 300 μm	R = 500 × 400 pixels, S1 = 14.5 × 10.5 mm^2^, S2 = 300 μm, t = 40 s; R = 10 × 8 pixels, S1 = 2.8 × 5 mm^2^, S2 = 300 μm, FPS = 16	[123,124]
	DLP-Based Digital Micromirror Device (DMD)	480 × 320 Micromirror Array; Modulation by Digital Switch	Si_3_N_4_/SiO_2_/Si/Au	Backside Illumination; f = 713 Hz, S = 4.3 μm	S1 = 20.8 × 15.6 mm^2^, S2 = 2.6 × 2.6 mm^2^, t = 2 s; S1 = 5 × 5 mm^2^, S2 = 0.87 × 0.87 mm^2^, t = 5 s; S1 = 1 × 1 mm^2^, S2 = 0.13 × 0.13 mm^2^, t = 60 s	[125,126]
	DLP-Based Projector	\	20 nm HfO_2_/1 μm a-Si/10 nm Mo/70 nm ITO/Glass	Backside Illumination; f = 30 Hz, S = 72 μm × 72 μm	R = 160 ×25 pixels, S1 = 2.88 × 1.8 mm^2^, S2 = 155× 155 μm^2^, t = 800 s; R = 98 × 22 pixels, S1 = 1.764 × 1.188 mm^2^, S2 = 106× 106 μm^2^, t = 431.2 s;	[42]
	OLED Display	High Modulation Frequency	Si_3_N_4_/SiO_2_/Si/Al	Backside Illumination; f = 1.74 kHz, S = 200 × 200 μm	R = 96 × 64 pixels, S1 = 20.1 × 13.2 mm^2^, S2 = 0.4 × 0.2 mm^2^, t = 150 s	[129]
Multi-Frequency Modulation Light Source Array (FDM)	High-Density VCSEL Array	12 VCSEL Diodes with a Pitch of 250 μm	Ta_2_O_5_/SiO_2_/Si	Backside Illumination; λ = 850 nm, f = 3 kHz ~ 4.1 kHz, Step = 100 Hz, S = 500 μm	R = 12 × 22 pixels, S1 = 3 × 10 mm^2^, S2 = 0.5 mm × 3 mm, t = 3.6 s	[133]
	Two-Dimensional LED Array	7 × 5 LED Array; Illumination Line by Line	100 nm Si_3_N_4_/50 nm SiO_2_/200 μm Si	Frontside Illumination; λ = 660 nm, f = 6 ~ 10 kHz, Step = 1 kHz, S = 2 mm	R = 7 × 5 pixels, S1 = 17 × 12 mm^2^, S2 = 2 mm × 12 mm, FPS = 70	[134]
	Optical Fiber Array with Microfluidic Channel	64 Light Beams; Flexible Measurement Layout	PDMS/Si_3_N_4_/SiO_2_/Si	Frontside Illumination; λ = 600 ~ 625 nm, f = 6.4 ~ 12.7 kHz, Step = 100 Hz, S = 500 μm	R = 8 × 8 pixels, S1 = 12 × 12 mm^2^, S2 = 0.5 mm × 0.5 mm, FPS = 100	[131]

^1^ λ: Wavelength; f: Frequency; S: Light spot size. ^2^ R: Image resolution; S1: Image area; S2: Measurement spot size; t: Imaging time; FPS: Frames per second.
